# Correlation between RVOT sizing and RV function and volumes in patients with repaired tetralogy of Fallot undergoing routine CMR follow-up: is there a better candidate for percutaneous pulmonary valve implantation?

**DOI:** 10.1186/1532-429X-17-S1-P209

**Published:** 2015-02-03

**Authors:** Aurelio Secinaro, Giuseppe Muscogiuri, Marcello Chinali, Benedetta Leonardi, Gabriele Rinelli, Giacomo Pongiglione

**Affiliations:** Bambino Gesù Children’s Hospital, Rome, Italy

## Background

CMR is the established non-invasive tool for routine RV functional and morphological assessment in repaired tetralogy of Fallot (rTOF) patients. 3D anatomical data of the RVOT are routinely performed to better address indications for Pulmonary Valve Replacement (PVR), either surgical or percutaneous. The aim of this cross-sectional analysis is to improve the ability of CMR to identify patients suitable for percutaneous pulmonary valve implantation (PPVI).

## Methods

From June 2012 to March 2014 patients with rToF (transannular and/or infundibular patch) and significant pulmonary regurgitation (PR) underwent routine CMR for RV assessment, including RV volumes (absolute and indexed RVEDV, RVESV) and ejection fraction (RVEF). In a sub-group of 31 patients a targeted 3D SSFP navigator sequence set at end-systole (trigger delay 270-310 ms) was also performed to better assess the pulmonary trunk (PT) morphology, length and dimensions. Transverse Diameter (TD) and Superior-Inferior Diameter (SID) and area obtained from the 3D dataset according to vessel analysis principles at three levels (PV remnant, mid-portion, bifurcation). Current eligibility criterion for percutaneous treatment (PPVI) is a maximum PT diameter up to 27 mm. We assumed that after PPVI the geometry of the PT will shift from elliptic to circular, thus we calculated a predicted PT {pPT= sqrt(4 * elliptic area / pi)} circular diameter through geometrical correction of the measured elliptic area at the PV remnant level.

Statistical analysis was performed using Spearman's correlation, univariate linear regression analysis and receiver operating charachteristic (ROC) curve.

## Results

A statistical significant positive correlation was observed between EDV, the area and TD of pulmonary remnant (p < 0,01), even if no correlation was observed between EDV and DSI.

No correlation was observed between SID, TD, the area and RV ESV and RVEF.

**Figure 1:**
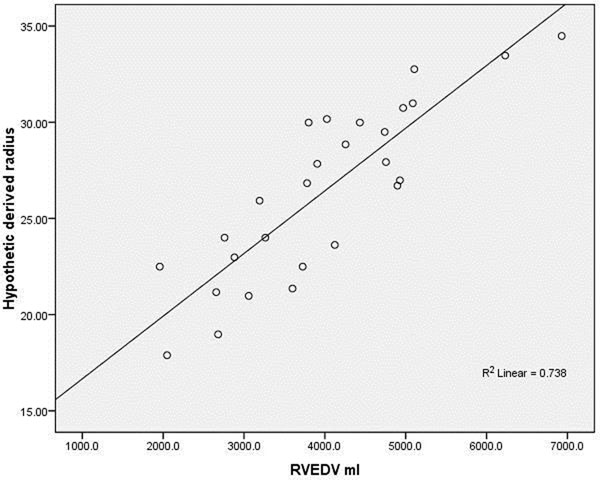
Plot representing the correlation between pPT and EDV.

The pPT diameter showed a stronger correlation to EDV (both absolute and indexed) as compared to individual observed PT diameters (R^2^= 0.74 - Fig. 1).

When comparing the current anatomical criteria for PPVI eligibility to the pPT diameter the number of eligible patient increased from 3 to 11 (p<0.05). In addition, in ROC analysis an EDVi between 134 and 139mL/m2 best identified patients eligible for PPVI according to the pPT diameter (AUC 0,44).

## Conclusions

Although the limited number of patients our study suggests that geometrically predicted PT diameters is more strongly associated with RV EDV as compared to measures currently used in clinical practice. In addition, use of predicted PT diameter significantly improves the identification of PPVI eligible patient. Our study also suggests that higher rates of PPVI eligible patients are present when EDVi is between 134 and 139mL/m^2^.

